# Interactive role of miR‐29, miR‐93, miR‐205, and VEGF in salivary adenoid cystic carcinoma

**DOI:** 10.1002/cre2.678

**Published:** 2022-10-25

**Authors:** Parisa Bayat, Nazanin Mahdavi, Shima Younespour, Neda Kardouni Khoozestani

**Affiliations:** ^1^ School of Dentistry, Dentistry Research Institute Tehran University of Medical Sciences Tehran Iran; ^2^ Department of Oral and Maxillofacial Pathology, School of Dentistry Tehran University of Medical Sciences Tehran Iran; ^3^ Cancer Institute, Imam Khomeini Hospital Complex Tehran University of Medical Sciences Tehran Iran

**Keywords:** miR‐205, miR‐29, miR‐93, salivary adenoid cystic carcinoma

## Abstract

**Objectives:**

Salivary adenoid cystic carcinoma (SACC) is one of the most common salivary gland tumors in which patients encounter local recurrence and lung metastases. Understanding prognostic biomarkers in SACC is essential for future development in prognosis and treatment. This study aimed to assess the expression level of vascular endothelial growth factor (VEGF) and its potential regulatory microRNAs in SACC for prognostic determination.

**Material and Methods:**

The expression of VEGF in SACC samples was assessed using immunohistochemistry. Potential regulatory microRNAs were evaluated using quantitative reverse transcription‐polymerase chain reaction. Associations between VEGF and microRNAs expression and clinicopathological parameters were investigated.

**Results:**

VEGF expression levels positively correlated with histologic grade (*p* = .004) and treatment modality (*p* = .04). Decreased expression of miR‐29a (*p* = .01) and increased expression of miR‐93‐5p and miR‐205 (both *p* < .0001) were observed in SACC compared to normal salivary gland tissue. MiR‐93‐5p showed a positive association (*p* = .02) with VEGF overexpression.

**Conclusions:**

Our results showed the downregulation of miR‐29 and overexpression of miR‐93 and miR‐205 in the SACC group, and the correlation between miR‐93 and VEGF suggests these biomarkers as potential prognostic markers in the future.

## 1 INTRODUCTION

According to the Global Cancer Observatory, salivary gland carcinomas (SGC) are comparatively rare tumors with an annual worldwide incidence of 0.07% and 0.05% in males and females, respectively, corresponding to 53,583 new cases per year in 2020 (Sung et al., 2021).

Salivary adenoid cystic carcinoma (SACC) is among the most common SGCs, accounting for approximately 10% of salivary gland neoplasms (El‐Naggar et al., 2017). SACC is a slow‐growing biphasic salivary gland malignancy of both major and minor salivary glands with neoplastic cells of epithelial and myoepithelial origin, which are classified according to their histologic patterns into tubular, cribriform, and solid forms (Brown et al., 2019; El‐Naggar et al., 2017). Despite the recent improvement in surgical resection associated with radiotherapy with or without chemotherapy for SACC, more than one‐third of the patients yet encounter local recurrence and lung metastases (Andreasen et al., 2018; Xie et al., 2018). Moreover, the overall survival has not been enhanced due to distinctive slow‐growing patterns, neurotropic infiltrative growth, local recurrence, and hematogenous spread (Jiang et al., 2015a; Xie et al., 2018).

The vascular endothelial growth factor (VEGF) family is a well‐known factor that regulates angiogenesis, and VEGF‐A acts as an essential member of the VEGF family in migration, growth, and angiogenic pathways (E. Dos Santos, Ramos, et al., 2021). Overexpression of VEGF is predicted to be targeted by multiple microRNAs (Kwon et al., 2018).

MicroRNAs (miRNAs) are noncoding RNAs (19–25 nucleotides in length) regulating gene expression after transcription by causing targeted messenger RNA (mRNA) degradation or translation inhibition, and this results in regulating various physiologic activities such as metabolism, development, and differentiation (E. S. Dos Santos, Normando, et al., 2021). They also play a crucial role in different steps of tumorigenesis, including tumor initiation and progression, by being involved in signaling pathways as either oncogenic factors or tumor suppressors (Cinpolat et al., 2017; E. S. Dos Santos, Normando, et al., 2021).

Among salivary gland malignancies, SACC is the most studied one for the possible role of miRNAs in the pathogenesis of cancers (Cinpolat et al., 2017). However, the potential regulatory molecular mechanisms underlying SACC are yet to be defined. Further efforts are required to uncover these mechanisms, which may provide a better therapeutic outcome in the future. MiR‐29 has been considered a tumor suppressor in numerous cancers, including lung and breast cancer (Kwon et al., 2018). Moreover, in pleomorphic adenoma and adenoid cystic carcinoma of the salivary gland, downregulation of MiR‐29 has been reported (Flores et al., 2017; Mitani et al., 2013). Accumulating studies have shown miR‐93 as an oncogenic factor in renal cell carcinoma and gastric cancer (Du & Kong, 2021; Guan et al., 2017). Also, in head and neck region cancers such as laryngeal carcinoma, it has been reported as an oncomiR, and there is a study on SACC showing that it upregulates as well (Mitani et al., 2013; Popov et al., 2020). Numerous studies investigated the role of miR‐205, and it is reported to be a tumor suppressor in ovarian and hepatocellular carcinomas (Lu et al., 2018; Qiao et al., 2020). In head and neck squamous cell carcinoma, miR‐205 has been proved to be a downregulating miR (Salazar‐Ruales et al., 2018). However, miR‐205 was thoroughly expressed in the primary SACC (Feng et al., 2017).

In conclusion, the precise mechanisms of miRNAs in emerging studies have not been fully elucidated. To discover the hidden mechanisms of tumor progression and poor prognosis, discovering the hidden molecular mechanism is unavoidable (Xu et al., 2020). Thus, this study is aimed to evaluate the role of the mentioned miRNAs and VEGF in SACC.

## 2 MATERIALS AND METHODS

### 2.1 Ethics approval

This study was approved by the Research Ethics Committee of Tehran University of Medical Sciences (IR.TUMS.DENTISTRY.REC.1400.043). Written informed consent was obtained from all patients before participation.

### 2.2 Case selection

Thirty paraffin‐embedded samples were collected, including 15 SACC and 15 normal salivary gland samples. We derived SACC samples from patients diagnosed with primary SACC in major and minor salivary glands between 2013 and 2019 and had surgery at the cancer institute of Imam Khomeini Hospital (Tehran University of Medical Sciences (TUMS), Tehran, Iran). Hematoxylin and eosin (H&E)‐stained slides for each patient were re‐evaluated to confirm the pathologic diagnosis of SACC, and available clinicopathologic data were gathered. Samples were histologically evaluated and graded according to the World Health Organization classification of tumors (El‐Naggar et al., 2017). Also, the clinical staging is based on the 8th edition of the American Joint Committee on Cancer staging system (Lydiatt et al., 2017). Patients with a history of chemotherapy, radiotherapy, or any other types of cancer before SACC were excluded. According to the patients’ self‐report, all the patients were nonsmokers and without any medical condition other than diagnosed SACC. All the control subjects were obtained for the purpose of misdiagnosing salivary gland tumors, or they were collected from patients with clinical differential diagnosis including Sialolithiasis or Sjogren's syndrome, which were diagnosed as normal after removal. All the samples were evaluated again and diagnosed as healthy control tissues. Demographics and clinicopathological features of total patients of this study are given in Table 1.

**Table 1:** Baseline demographics and clinicopathological characteristics of the study groups

Characteristic	SACC (*n* = 15)	Control (*n* = 15)	*p* Value
Age	56.07 ± 13.52	56.40 ± 12.61	.94
Gender			.71
Female	6 (40.00%)	7 (46.67%)	
Male	9 (60.00%)	8 (53.33%)	
Anatomic site			.06
Major salivary glands	7 (46.67%)	12 (80.00%)	
Parotid	5 (33.33%)	8 (53.33%)	
Submandibular	2 (13.33%)	1 (6.67%)	
Sublingual	‐	3 (20.00%)	
Minor salivary glands	8 (53.33%)	3 (20.00%)	
Hard palate	7 (46.67%)	3 (20.00%)	
Mandible	1 (6.67%)	‐	
Tumor size in greatest dimension			
Median (IQR), range	5.70 (1.80–8.00), (1.20–9.00)	‐	
Histopathological grades			‐
Grade I	5 (33.33%)	‐	
Grade II	6 (40.00%)	‐	
Grade III	4 (26.67%)	‐	
Stage			
Stage I	3 (20.00%)	‐	
Stage II	2 (13.33%)	‐	
Stage III	8 (53.33%)	‐	
Stage IVa	2 (13.33%)	‐	
Lymphovascular invasion	5 (33.33%)	‐	
Perineural invasion	10 (66.67%)	‐	
Treatment			
Total parotidectomy	4 (26.67%)	‐	
Partial parotidectomy	1 (6.67%)	‐	
Hard palate excision	5 (33.33%)	‐	
Submandibular gland excision	1 (6.67%)	‐	
Mandibulectomy	2 (13.33%)	‐	
Hemimaxillectomy	2 (13.33%)	‐	

*Note*: Data are expressed as mean ± SD or no. (%), unless otherwise stated.

Abbreviation: SACC, salivary adenoid cystic carcinoma.

### 2.3 Bioinformatics assessments to identify suitable microRNAs

First, we uploaded miR‐29, miR‐205, and miR‐93 separately to the Targetscan database (http://www.targetscan.org/vert_80/) and mapped the common genes between the candidate miRs using Venn software version 2.1.2. Second, we used the Enrichr Dieter Reactome database (https://reactome.org/) to examine the signaling pathway for a closer look at the genes in common with mentioned regulatory miRs. Third, we used the GeneMania database (https://genemania.org/) to map the network to evaluate the VEGF‐A gene more accurately with other upstream and downstream proteins. Finally, using the miRTargetlinker database (https://ccb-web.cs.uni-saarland.de/mirtargetlink/), we further confirm the association of the three miRs with the VEGF gene. Thirty‐four genes are involved in essential signaling pathways such as PI3K/AKT, transcription factors, and metabolic signaling pathways. VEGF‐A is present and expressed in most of these signaling pathways. Also, other miRNAs, such as miR‐185, miR‐196, and miR‐372, regulate VEGF‐A gene expression. FLT1, KDR, PGF, and ARNT proteins are also closely related to the VEGF‐A gene.

### 2.4 Total RNA extraction and complementary DNA (DNA) synthesis

H&E‐stained slides of SACC samples were evaluated using a light microscope to determine the tumor‐containing areas. The 30 formalin‐fixed, paraffin‐embedded samples were incubated in xylene to eliminate the paraffin. Following a wash with ethanol, total RNA from our study subjects was isolated using the RiboEX RNA Extraction Kit (GeneAll, Seoul, South Korea) according to the manufacturer's protocols. DNase I was used to treat extracted RNA to achieve the final product with no contamination of the genomic DNA and stored at −80°C. The purity and concentration of extracted RNA were evaluated by measuring the absorbance of OD260, and OD260/OD280 ratio was calculated to assess the quality and quantity of isolated RNA using Thermo Scientific NanoDrop 2000. Next, the extracted RNA was diluted to 1 g/µl. Afterward, Thermo Scientific RevertAid First Strand cDNA Synthesis Kit (Thermo Fisher Scientific, Waltham, MA, USA) was used to make a reverse transcription of the total RNA to single‐stranded cDNA.

### 2.5 Quantitative real‐time PCR

Primer 3 plus was used to design the primers of the VEGF gene, miR‐29a, miR‐93‐5p, and miR‐205. The Real‐Time PCR assay was carried out by applying the gene‐specific primers SYBR green technique using BioFACT 2X Real‐Time PCR Master Mix (BioFACT, Daejeon, South Korea). In addition, stem‐loop primers were used to assess the expression for microRNA assay in this study. Reference genes U6 (small nuclear RNA) and β‐Actin were used as an endogenous control to normalize the expression levels of microRNAs and mRNA, each recorded as threshold cycle numbers (*C*_t_), which is defined as the number of cycles required for the fluorescent signal to cross the threshold. Finally, using the $2^{-\Delta \Delta C_{T}}$ formula, the final fold changes of VEGF, miR‐29, miR‐93, and miR‐205 expression levels were calculated.

### 2.6 Immunohistochemistry

The immunohistochemistry (IHC) method was used to detect VEGF in SACC samples. For each tumor and normal salivary gland tissue, 4‐μm formalin‐fixed paraffin‐embedded sections were obtained. We deparaffinized all sections in xylene and rehydrated them with gradient alcohol. The VEGF antibody (Medaysis, Liverm CA, USA) was applied according to the manufacturer's instructions. Placental tissue as a positive control was used to check the IHC process, and for the negative control, we substituted the primary antibody with nonimmune serum.

### 2.7 Immunohistochemical evaluation

The slides were observed separately by two pathologists who were blinded to clinical and pathologic information under a high‐resolution light microscope (BX40 Olympus, Tokyo, Japan) in SACC cases; 10 hotspot areas were chosen randomly to assess the VEGF antibody semiquantitatively. The proportion of positively stained SACC cells was graded from 0 to IV (0 = no positive cell; I = <25% of tumoral cells; II = 25%–50% of tumoral cells; III = 50%–75% of tumoral cells; IV = >75% of the tumoral cells. The intensity of staining was also measured on a scale of 0–3 (0 = *no staining*; +1 = *mild*; +2 = *moderate*; +3 = *intense*). The final score of each field was determined by combining the score of the intensity of staining and positive stained SACC cells. The sum of all 10 areas in each case was calculated and then divided into 10 to assess the mean to evaluate the final VEGF expression of every case (Faur et al., 2014). When the sum of the two obtained values was ≤2, it was considered as negative staining, and scores over 3 were categorized as positive staining and subcategorized as moderate (3–4) and strong (5–7).

### 2.8 Statistical analysis

Statistical analyses were performed with statistical software IBM SPSS Statistics for Windows, version 23 (IBM Corp., Armonk, NY, USA) and MedCalc® Statistical Software version 19.8 (MedCalc Software Ltd., Ostend, Belgium; https://www.medcalc.org; 2021). All tests were two‐sided, and *p* < .05 was considered statistically significant. The Shapiro–Wilk *W*‐test was used to check the normality assumption of the continuous variables. Independent‐sample *t*‐test or Mann–Whitney *U*‐test, whenever appropriate, was applied to test the differences between the means of two groups. The *χ* ^2^ test was used to examine the association between two categorical variables. Pearson or Spearman correlation tests were applied to assess the association between two variables.

## 3 RESULT

Patients with SACC and normal salivary gland samples were included in the current study. Baseline demographics and clinicopathological characteristics of the study groups are presented in Table 1. The two groups did not differ significantly in age, gender, and anatomic site (*p* = .94, *p* = .71, and *p* = .06, respectively; Table 1).

The mean follow‐up of SACC patients was 4.07 ± 1.67 years, ranging from 2 to 7 years. There were five deaths, of which three were due to cancer and two were unrelated to cancer.

### 3.1 VEGF immunohistochemical finding

**Figure 1:**
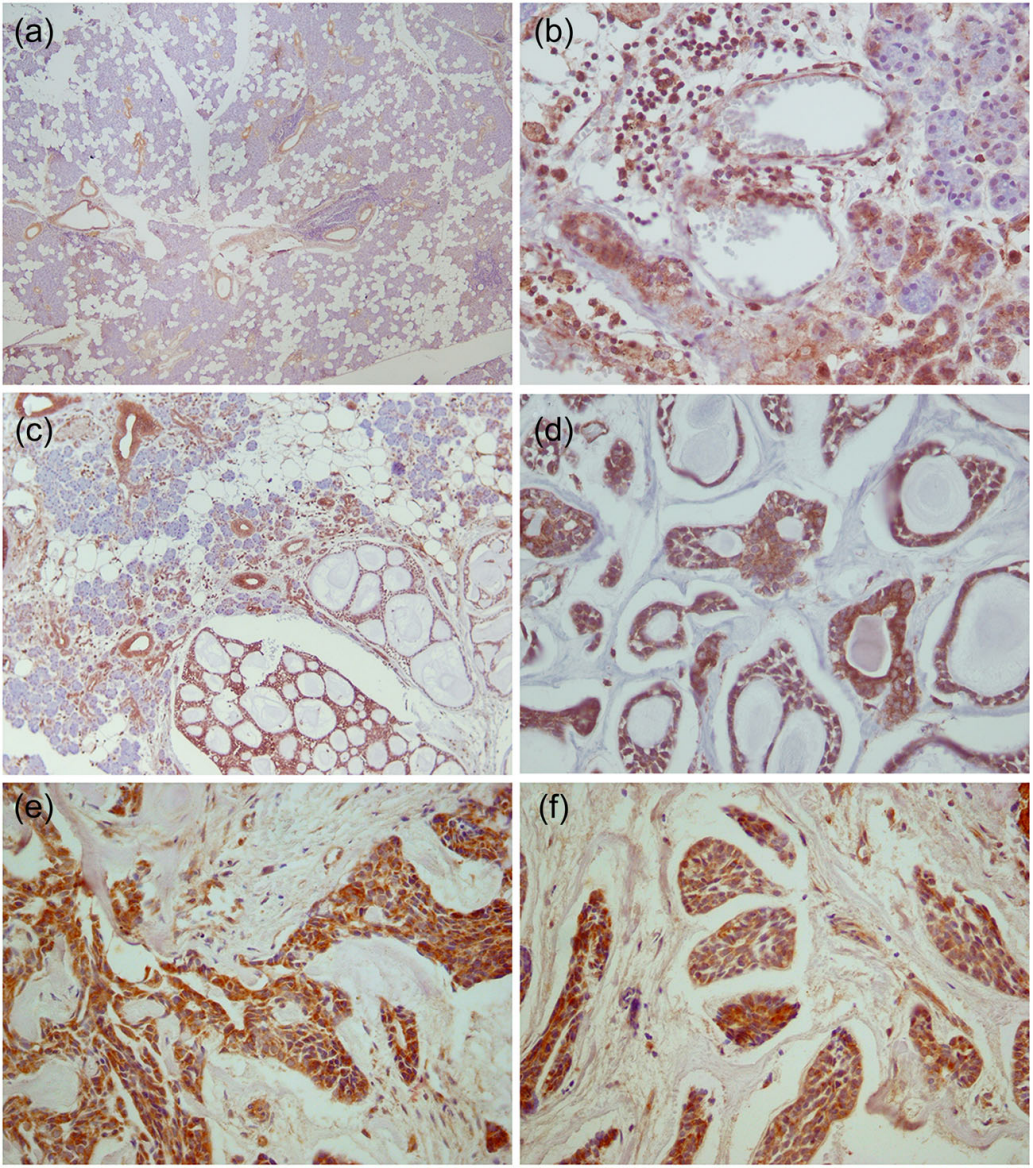
Vascular endothelial growth factor immunohistochemistry staining of SACC in normal serous salivary gland tissue (a), in inflammatory cells infiltration of an inflamed specimen (b), SACC cribriform pattern (c, d), SACC solid pattern (e, f). SACC, salivary adenoid cystic carcinoma.

Representative photomicrographs of normal salivary gland tissue and SACC tumor in intermediate and high grades are depicted in Figure 1a–f. Mainly cytoplasmic and less frequently membranous VEGF immunostaining of striated and intercalated ducts, myoepithelial, epithelial cells, and scattered mild to moderate granular cytoplasmic staining in the serous acinar cells were observed in normal tissue. Furthermore, in inflammatory cells, positive immunostaining was spotted in macrophages and lymphoplasma cells. Cytoplasmic staining in both epithelial and myoepithelial tumoral cell populations in a grade‐dependent manner was noted. Moderate to severe staining of VEGF was observed in the lining squamous epithelium of the mucosa. Based on the scoring, all the SACC samples were positive. The mean IHC expression level of VEGF was 5.33 ± 1.29. The mean IHC expression level of VEGF was 4.40 in patients with Grade I SACC, 5.16 in Grade II, and 6.75 in Grade III.

### 3.2 Association between the immunohistochemical expression level of VEGF and selected patient characteristics

A positive association was found between histopathologic grade and IHC expression level of VEGF in patients with SACC (*r* = .70, *p* = .004). There was a significant difference between radical and conservative treatment modalities regarding the IHC expression level of VEGF (*p* = .04). However, no significant association was found between this parameter and age (*p* = .31), gender (*p* = .69), anatomic site (*p* = .20), tumor size (*p* = .73), tumor stage (*p* = .14), lymphovascular invasion (*p* = .89), and perineural invasion (*p* = .88) (Table 2).

**Table 2:** Association between the SACC patient characteristics and both VEGF IHC score and relative expression levels of microRNAs

Characteristic	IHC level of VEGF		Relative expression levels of microRNAs					
	IRS	*p* Value	miR‐29a	*p* Value	miR‐93‐5p	*p* Value	miR‐205	*p* Value
Age	*r* = .28	.31	*r* = −.50	.06	*r* = −.33	.23	*r* = −.29	.29
Gender		.69		.39		.43		.77
Female	5.17 ± 1.17		0.74 ± 0.26		35.25 ± 13.89		24.99 ± 14.76	
Male	5.44 ± 1.42		0.62 ± 0.21		29.26 ± 13.97		27.41 ± 15.95	
Anatomic site		.20		.09		.61		.61
Major salivary glands	4.86 ± 1.34		0.78 ± 0.26		33.68 ± 13.04		28.60 ± 10.99	
Minor salivary glands	5.75 ± 1.64		0.56 ± 0.15		29.89 ± 15.03		24.54 ± 18.35	
Tumor size	*r* = .10	.73	*r* = .43	.11	*r* = .43	.11	*r* = .62	.01
Histopathologic grades	*r* = .70	.004	*r* = −.13	.64	*r* = .65	.01	*r* = .50	.06
Tumor stage	*r* = .14	.62	*r* = .09	.76	*r* = .44	.10	*r* = .46	.09
Lymphovascular invasion		.89		.97		.35		.52
Yes	5.40 ± 1.14		0.66 ± 0.22		36.43 ± 12.84		29.84 ± 12.69	
No	5.30 ± 1.42		0.67 ± 0.25		29.27 ± 14.24		24.74 ± 16.38	
Perineural invasion		.88		.01		.50		.42
Yes	5.30 ± 1.42		0.75 ± 0.24		33.64 ± 13.00		29.23 ± 12.31	
No	5.40 ± 1.14		0.50 ± 0.08		27.71 ± 15.95		20.86 ± 19.67	
Treatment modality		.04		.91		.62		.68
Conservative	4.33 ± 0.58		0.65 ± 0.22		29.35 ± 5.88		24.53 ± 5.04	
Radical	5.58 ± 1.31		0.67 ± 0.24		32.24 ± 15.27		26.92 ± 16.73	

*Note*: Data are expressed as mean ± SD; or Spearman's or Pearson correlation coefficient.

Abbreviations: IHC, immunohistochemistry; IRS, Immunoreactivity score; SACC, salivary adenoid cystic carcinoma; VEGF, vascular endothelial growth factor.

### 3.3 VEGF quantitative reverse transcription‐polymerase chain reaction (qRT‐PCR) and relative expression levels of microRNA findings

**Figure 2:**
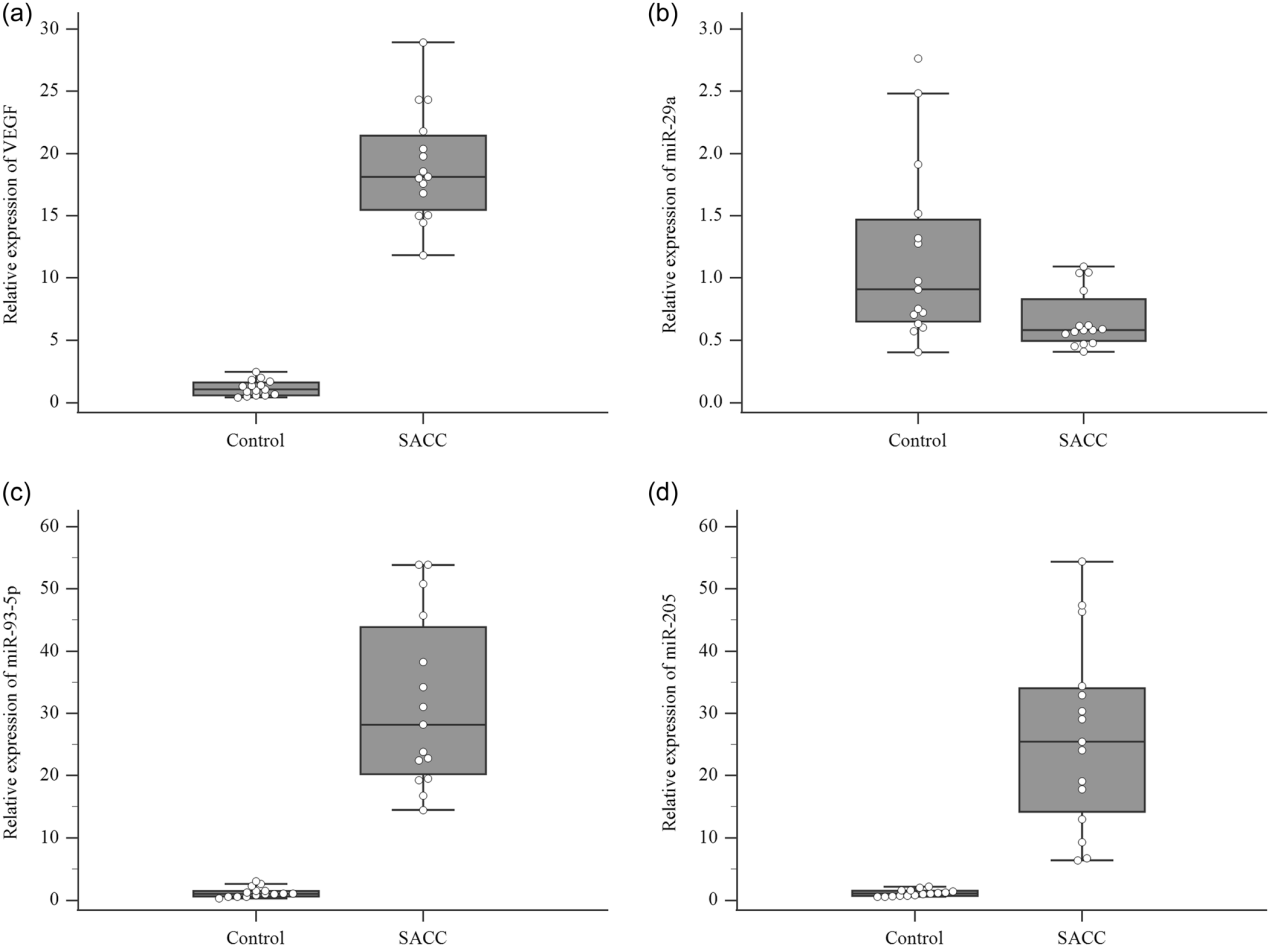
Relative expression levels of (a) VEGF, (b) miR‐29a, (c) miR‐93‐5p, and (d) miR‐205, Middle point: median; box: interquartile range (25th to 75th percentiles); whisker: range (excluding outliers). Each error bar is constructed using a 95% confidence interval of the mean. miR, microRNA; SACC, salivary adenoid cystic carcinoma; VEGF, vascular endothelial growth factor.

The mean expression level of VEGF was 18.99 ± 4.46 in the SACC group and 1.16 ± 0.62 in the control group. Higher expression levels of the VEGF gene were found in the SACC group in comparison with the control group (*p* < .0001 and Figure 2a).

Relative expression levels of microRNAs are summarized in Table 3. The median miR‐29a expression level was significantly lower in the SACC group than in normal salivary gland tissues (*p* = .01 and Figure 2b). The median miR‐93‐5p and miR‐205 expression levels were significantly higher in the SACC group compared to those of normal salivary gland tissues (both *p* < .0001 and Figure 2c,d).

**Table 3:** Relative expression levels of microRNAs and VEGF in SACC and control groups

	SACC (*n* = 15)	Control (*n* = 15)	*p* Value
miR‐29a			.01
Median (IQR), range	0.58 (0.48–0.90), (0.41–1.09)	0.91 (0.63–1.52), (0.40–2.76)	
Mean ± SD	0.66 ± 0.23	1.17 ± 0.72	
miR‐93‐5p			<.0001
Median (IQR), range	28.17 (19.51–45.76), (14.48–3.85)	0.99 (0.51–1.45), (0.26 –3.02)	
Mean ± SD	31.66 ± 13.77	1.22 ± 0.80	
miR‐205			<.0001
Median (IQR), range	25.46 (13.00–34.42), (6.39–54.38)	1.05 (0.65–1.52), (0.53–2.14)	
Mean ± SD	26.44 ± 14.99	1.10 ± 0.51	
VEGF			<.0001
Median (IQR), range	18.13 (15.03–21.78), (11.84–28.94)	1.05 (0.54–0.68), (0.40–2.45)	
Mean ± SD	18.99 ± 4.46	1.16 ± 0.62	

Abbreviations: IQR, interquartile range (25th to 75th percentiles); SACC, salivary adenoid cystic carcinoma; VEGF, vascular endothelial growth factor.

### 3.4 Association between relative expression levels of microRNAs and selected patient characteristics

A significant difference was observed between SACC patients with and without perineural invasion regarding the median relative expression level of miR‐29a (*p* = .01). There was a significant correlation between the relative expression level of miR‐93‐5p and histopathologic grade (*r* = .65 and *p* = .01). The relative expression level of miR‐205 was correlated with tumor size (*p* = .01). Complete data are summarized in Table 2.

### 3.5 Association between relative expression levels of microRNAs and immunohistochemical expression level of VEGF

There was a positive association between the relative expression level of miR‐93‐5p and the IHC expression level of VEGF (*r* = .59, *p* = .02). However, there was no significant correlation between IHC expression level of VEGF and both miR‐205 (*r* = .35, *p* = .20) and miR‐29a (*r* = −0.38, *p* = .16).

### 3.6 Association among relative expression levels of microRNAs

There was a positive association between relative expression levels of miR‐93‐5p and miR‐205 (*r* = .77, *p* = .001). However, no significant association was found between relative expression levels of miR‐93‐5p and miR‐29a (*r* = −.06, *p* = .83) and miR‐205 and miR‐29a (*r* = .22, *p* = .44).

## 4 DISCUSSION

Protein Coding VEGFA gene and regulatory microRNAs have critical roles in the diagnosis and prognosis of several neoplasms, including salivary gland tumors (E. S. Dos Santos, Normando, et al., 2021). VEGF is a potent proangiogenic factor that has been one of the most studied cancer biomarkers because of its role in angiogenesis and vasculogenesis. VEGF overexpression has been involved in microvascular permeability and tumor cell penetration, leading to invasion and metastasis of various tumors, including cervical and colorectal cancer (F. Wang et al., 2018).

Moreover, in the head and neck region, VEGF is considered as a prognostic factor in papillary thyroid carcinoma and oral tongue squamous cell carcinoma, where its overexpression relates to poor overall survival (F. Wang et al., 2018). Recent systematic review and meta‐analysis evaluating the role of VEGF in malignant salivary gland tumors depicted that this angiogenic factor has been associated with prognosis and tumor progression; however, in some malignant salivary gland tumors, the prognostic value of VEGF is yet unclear (E. Dos Santos, Ramos, et al., 2021).

For instance, an immunohistochemical study on 68 SACC cases revealed that poor survival is correlated with higher VEGF expression as an independent prognostic factor (Park et al., 2016). Furthermore, in a study designed for immunoexpression of VEGF in breast cancer subtypes, the result showed that higher expression of VEGF is associated with pathological grade (Liu et al., 2014). However, in the Fonseca experiment on 20 adenoid cystic carcinomas, there was no association between VEGF expression and the grade of the tumor (Fonseca et al., 2015).

In our experiments, using RT‐PCR, we found higher expression levels of the VEGF gene in the SACC group compared to the control group. In line with Liu's study, we also found a significant positive association between the IHC expression level of VEGF and grade in patients with SACC. We also noted the highest VEGF expression levels in three patients who passed away because of SACC among all cases.

Over the past years, miRNAs have attracted much attention among scientists as the most studied gene posttranscriptional regulators modulating tumors by affecting their possible target genes (Rupaimoole et al., 2016). In head and neck cancers, 22 miRs were the most studied ones, including miR‐29 in the nasopharynx, as well as miR‐93 and miR‐205 in head and neck squamous cell carcinomas (HNSCCs) (E. S. Dos Santos, Normando, et al., 2021; Patil & Warnakulasuriya, 2020).

MiR‐93 is a tumor oncogenic microRNA that has been associated with ophthalmological studies focusing on diabetic retinopathy or glaucoma via experimenting on VEGF‐related pathways (Fuchs et al., 2020). In a study on miR‐93 and tumor growth factor‐β‐mediated epithelial–mesenchymal transition (EMT) pathway on arising retinal pigment epithelium‐19 (ARPE‐19) cells, EMT was found to be triggered in mesenchymal ARPE‐19 to epithelial transition (Fuchs et al., 2020). Moreover, a study on Type 2 diabetic retinopathy using qRT‐PCR and Western blot analysis demonstrated that while increasing the activity of antioxidative indicators, miR‐93‐5p inhibition downregulated VEGF levels and proinflammatory cytokines via Sirt1 overexpression, possibly through YAP/HIF1a/VEGFA pathway (H. Wang et al., 2021). MiR‐93 in glioma cell lines and glioma tissues was significantly upregulated, and it is correlated with clinicopathologic grading and survival in patients and may directly activate PI3K/Akt signaling by suppressing PTEN, PHLPP2, and FOXO3 expression targeting 3′‐untranslated regions (UTRs) (Jiang et al., 2015b). In the head and neck region, a study evaluating miR‐93 in advanced laryngeal carcinoma using microRNA global profiling suggested that miR‐93 is a tumor promoter via targeting VEGF directly (Popov et al., 2020). Also, a study on HNSCC samples using qRT‐PCR demonstrated significantly higher expression of miR‐93. It was correlated with poor prognosis, tumor progression, and metastasis, indicating miR‐93 as a valuable marker in HNSCC (Li et al., 2015). In agreement with many of mentioned studies, we found a higher expression of miR‐93 in SACC compared to normal salivary gland tissue, as well as confirming a positive correlation between miR‐93 expression level with VEGF expression and SACC grade. This finding confirms the oncogenic role of miR‐93 in VEGF‐related pathways, possibly via PI3K/Akt signaling pathway, as suggested in numerous articles. On the other hand, few studies indicated miR‐93 as a tumor suppressor factor, including a study using qRT‐PCR in neuroblastoma, which revealed that the 3′‐UTR region of VEGF mRNA is the miR‐93 molecular target in SK‐N‐AS cell line as miR‐93 plays a role as a tumor suppressor miR (Fabbri et al., 2016).

MiR‐29 has been studied in multiple cancers, including breast and lung cancer, suggesting a tumor suppressor role resulting in proliferation and migration (Kwon et al., 2018). In addition, miR‐29 via targeting VEGF in PI3K/AKT and JAK/STAT signaling pathways in nasopharyngeal carcinoma 5‐8F cells using qRT‐PCR and Western blot showed suppression by increasing invasion, cells growth, migration, tumor cells proliferation, and angiogenesis via decreased phosphorylation levels of AKT, JAK1, STAT1, and STAT3 (Shi et al., 2019). Unlike the previous studies, the expression of miR‐29a in osteosarcoma has been evaluated, indicating that IGF1‐30UTR functions as a competing endogenous RNA in increasing angiogenesis by sponging miR‐29 (Gao et al., 2016). In salivary gland tumors, namely, pleomorphic adenoma and adenoid cystic carcinoma of the salivary gland, downregulation of miR‐29 has been reported (Flores et al., 2017; Mitani et al., 2013). However, there are no studies evaluating the correlation between miR‐29 and angiogenesis and survival.

The current study showed a lower expression level of miR‐29 in SACC patients. Due to limitations, we failed to estimate a statistically significant relation between VEGF and miR‐29. However, there is a negative relation between miR‐29 and VEGF expression, suggesting tumor suppressor role for miR‐29m probably via PI3K/AKT and JAK/STAT pathways as these are the most studied pathways.

MiR‐205 is involved in invasion, migration, and angiogenesis, including qRT‐PCR and Western blot analysis results on cervical and renal cancer, showing that miR‐205 activates the Akt signaling pathway (Huang et al., 2019; F. Zhang et al., 2019). In 109 paraffin‐embedded gastric cancer samples using qRT‐PCR, it is suggested that miR‐205 is involved in the induction of neovascularization via suppressing VEGF in the ERK signaling pathway. Moreover, miR‑205 in renal cancer was found to regulate VEGF via PI3K/Akt signaling pathway by several possible mechanisms. In oral squamous cell carcinoma, miR‐205 inhibits TIMP‑2 expression to suppress invasiveness (J. Zhang et al., 2021). However, in SACC, miR‐205 showed higher gene expression as a tumor oncogene (Feng et al., 2017). The current study showed a higher expression level of miR‐205 in SACC patients in line with previous SACC studies, suggesting a possible role of miR‐205 as a tumor oncogene. There was no significant positive relationship between miR‐205 and VEGF expression despite the increasing trend in both factors.

Both miR‐93 and miR‐205 regulate PTEN in the Akt pathway, one of the significant pathways, and overexpression of these miRs targets PTEN directly (Fu et al., 2012). Due to the limitations of our study, we did not investigate the hidden underlying mechanisms sharing between studied miRs. However, we found a positive association between expression levels of miR‐93 and miR‐205.

Taken together, our study brings out the novel findings of microRNAs related to VEGF signaling pathways in SACC, which is a less discussed tumor among other salivary gland malignancies despite accounting for 10% of total salivary gland neoplasms. Similar to that, the role of VEGF in salivary gland tumors, specifically SACC, is still hidden. Our study uncovers the role of VEGF in SACC by exploring this factor via IHC as it shows a positive association between histopathologic grade and IHC expression level of VEGF. We explored the role of three microRNAs in SACC individually in order to find out if they play as oncomiRs or tumor suppressors. In addition, we found that there is a positive association between the relative expression level of miR‐93‐5p and VEGF.

However, some limitations exist in the current study. Unfortunately, this study does not further discuss in what exact pathway the miR‐93 was positively correlated with VEGF. Application of miRs mimics and inhibitors is required to determine accurate signaling pathways. Another limitation of the study is that only three microRNAs were evaluated. Hence, more studies are suggested to assess other miRNAs in VEGF‐related pathways. In our study, we focused on one of the angiogenic factors (VEGF) and its relationship with microRNAs, and our study did not measure inflammation as a variable involved in possible microRNA pathways. However, we suggest investigating further studies on mapping possible microRNAs in order to target their roles in various inflammation pathways in common salivary gland malignancies. We also suggest survival analysis in future studies for better understanding.

In conclusion, our findings indicated that VEGF immunohistochemical overexpression was prominent in high‐grade SACC. MiR‐93 and miR‐205 as oncomiR microRNAs might be correlated with VEGFA. MiR‐29a as a tumor suppressor may associate with negative regulation of VEGF. The mentioned miRs add insights to our understanding of critical epigenetic modulators of angiogenesis in SACC and expand our perception of the related molecular state. In the future, these microRNAs may be used as biomarkers and therapeutic targets in SACC.

## AUTHOR CONTRIBUTIONS

All authors contributed to the conceptualization and design of the study. Laboratory procedures and data collection were performed by Neda Kardouni Khoozestani and Parisa Bayat. Results were analyzed and interpreted by Shima Younespour and Neda Kardouni Khoozestani. The draft of the manuscript was written by Parisa Bayat. Neda Kardouni Khoozestani and Nazanin Mahdavi wrote, reviewed, and edited the manuscript. All authors read and approved the final manuscript.

## CONFLICT OF INTEREST

The authors declare no conflict of interest.

## Supporting information

Supporting information.

## Data Availability

Data sets generated and analyzed during the study are available from the corresponding author upon reasonable request.
