# Supplementary material for: Interactive role of miR‐29, miR‐93, miR‐205, and VEGF in salivary adenoid cystic carcinoma
Source: Clin Exp Dent Res. 2022 Oct 25;9(1):112–21. doi: 10.1002/cre2.678 (PMC9932236; doi:10.1002/cre2.678)
Supplement: Supplementary file 1 — Supporting information. [file CRE2-9-112-s001.pdf]

Title: The Interactive role of miR-29, miR-93, miR-205, and VEGF in Salivary Adenoid Cystic Carcinoma

(Clinical and Experimental Dental Research Journal)

Parisa Bayat[1], Nazanin Mahdavi[2], Shima Younespour[3], Neda Kardouni Khoozestani*[2,4]

[1] Dental Research Center, Dentistry Research Institute, School of Dentistry, Tehran University of Medical Sciences, Tehran, Iran.
[2] Department of Oral and Maxillofacial Pathology, School of Dentistry, Tehran University of Medical Sciences, Tehran, Iran.
[3] Dentistry Research Institute, School of Dentistry, Tehran University of Medical Sciences, Tehran, Iran.
[4] Cancer Institute, Imam Khomeini Hospital Complex, Tehran University of Medical Sciences, Tehran, Iran.

***Corresponding author;**
Neda Kardouni Khoozestani
nkardouni@tums.ac.ir
Postal address: Dental School of Tehran University of Medical Sciences, North Kargar St, Tehran, Iran.
Phone: +982188351163

The supplementary materials include 1 supplementary figure and 2 supplementary tables

Fig. S1 Analysis and mapping of strongly associated miRNAs involved in regulating VEGF-A gene expression

Table S1 Primers' oligonucleotide sequences

Table S2 Demographic, clinicopathological characteristics, immunohistochemical and qRT-PCR VEGF expression level and relative expression levels of microRNAs of SACC patients who passed away related to SACC

Fig. S1 Analysis and mapping of strongly associated miRNAs involved in regulating VEGF-A gene expression

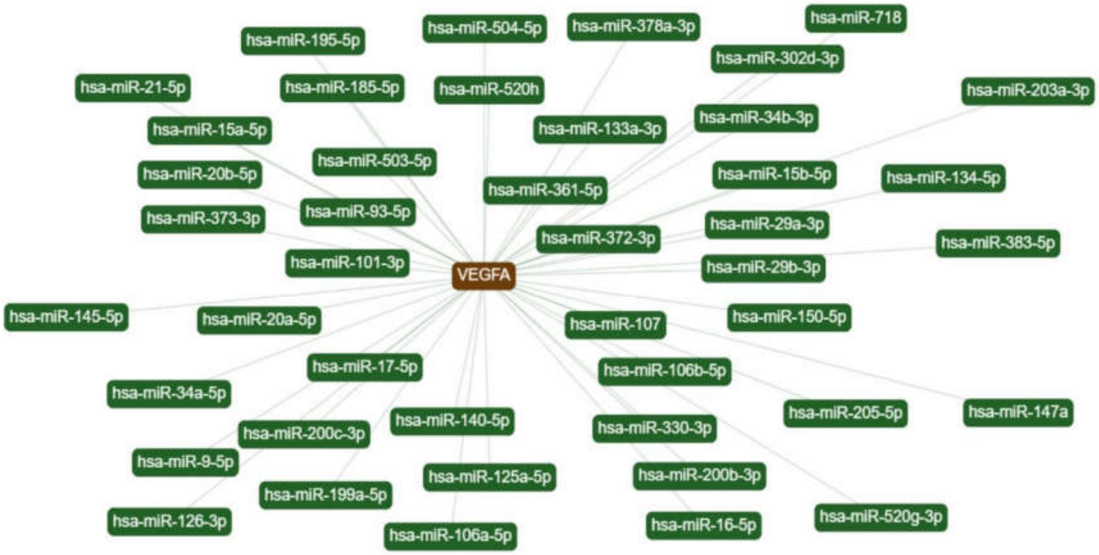

Table S1 Primers' oligonucleotide sequences

Stem-loop quantitative Real-Time PCR primer sequences	miR-29a	SL	GTCGTATCCAGTGCAGGGTCCGAGGTATTCGCACTGGATACGATAACCG
		FW	CGTAGCACCATCTGAAATCG
		RV	GTGCAGGGTCCGAGGT
	miR-93-5p	SL	GTCGTATCCAGTGCAGGGTCCGAGGTATTCGCACTGGATACGACCTACCT
		FW	AGTCTCTGGCTGACTACATCACAG
		RV	CTACTCACAAAACAGGAGTGGAATC
	miR-205	SL	GTCGTATCCAGTGCAGGGTCCGAGGTGCACTGGATACGACGAACAGA
		FW	TGCGGCCCAGTGTTTAGACTATC
		RV	CCAGTGCAGGGTCCGAGGT
	U6	SL	GTCGTATCCAGTGCAGGGTCCGAGGTATTCGCACTGGATACGACAAAATG
		FW	GCGCGTCGTGAAGCGTTC
		RV	GTGCAGGGTCCGAGGT
RT PCR primer sequences	VEGF	FW	TTGCCTTGCTGCTCTACCTCCA
		RV	GATGGCAGTAGCTGCGCTGATA
	ACTB	FW	CTGGAACGGTGAAGGTGACA
		RV	AAGGGACTTCCTGTAACAATGCA

Table S2 Demographic, clinicopathological characteristics, immunohistochemical and qRT-PCR VEGF expression level and relative expression levels of microRNAs of SACC patients who passed away related to SACC

Characteristics	Patient 1	Patient 2	Patient3
**Age, year**	60	60	72
**Gender**	Male	Male	Male
**Anatomic site**	Hard palate	Hard palate	Mandible
**Tumor size**	8.2	6	2.5
**Histopathologic grades**	III	III	III
**Tumor stage**	III	IVa	III
**Lymphovascular invasion**	Yes	No	No
**Perineural invasion**	Yes	No	Yes
**Treatment modality***	Hard palate excision	Hard palate excision	Mandibulectomy
**IHC expression level of VEGF**	7	7	7
**Relative expression level of VEGF**	14.42	18.57	21.78
**miR-29a**	0.45	0.41	0.55
**miR-93-5p**	50.77	53.85	23.85
**miR-205**	47.34	54.38	9.32
**Follow-up, years**	6	5	6
**Survival time**	3	2	4

Abbreviations: SACC, Salivary Adenoid Cystic Carcinoma; IHC, Immunohistochemical; VEGF, Vascular endothelial growth factor
